# ANALYSIS OF THE DIFFERENCES BETWEEN THE PRESCRIBED AND THE ADMINISTERED DIET TO PRETERM INFANTS USING AN ELECTRONIC TOO

**DOI:** 10.1590/1984-0462/;2019;37;4;00008

**Published:** 2019-07-18

**Authors:** Olivia Araújo Zin, Fernanda Valente Mendes Soares, Andrea Dunshee de Abranches, Ana Carolina Carioca da Costa, Letícia Duarte Villela, Maria Elisabeth Lopes Moreira

**Affiliations:** a Instituto Fernando Figueira, Fundação Oswaldo Cruz, Rio de Janeiro, RJ, Brazil.

**Keywords:** Nutrition therapy, Premature newborn, Electronic health records, Terapia nutricional, Recém-nascido prematuro, Registros eletrônicos de saúde

## Abstract

**Objective:**

To create an electronic instrument in order to analyze the adequacy of the preterm infants’ nutritional therapy, checking the difference between the prescribed and the administered diet.

**Methods:**

A prospective and observational study on newborns with birthweight ≤1,500g and/or gestational age ≤32 weeks, without congenital malformations. The electronic instrument was developed based on Microsoft Excel 2010 spreadsheets and aimed at automatically calculating body weight gain, calories and macronutrients received daily by each patient from parenteral nutrition, intravenous hydration and enteral feedings. The weekly means of each nutrient were used to compare the prescribed and administered diets.

**Results:**

To evaluate the instrument, 60 newborns with a birth weight of 1,289±305 g and a gestational age of 30±2 weeks were included. Of them, 9.6% had restricted growth at birth and 55% at discharge. The median length of stay was 45±17 days. There were significant differences between prescribed and administered diet for all of the macronutrients and for total calories in the first three weeks. The lipid was the macronutrient with the greatest percentage error in the first week of life.

**Conclusions:**

The use of a computational routine was important to verify differences between the prescribed and the administered diet. This analysis is necessary to minimize calculation errors and to speed up health providers’ decisions about the nutritional approach, which can contribute to patients’ safety and to good nutritional practice. Very low birth weight infants are extremely vulnerable to nutritional deficiencies and any reduction in macronutrients they receive may be harmful to achieve satisfactory growth.

## RESUMO

**Objetivo:**

Elaborar um instrumento eletrônico para análise da adequação da terapia nutricional dos recém-nascidos pré-termo, verificando a diferença entre a dieta prescrita e a administrada.

**Métodos:**

Estudo observacional prospectivo em recém-nascidos com peso de nascimento ≤1.500 g e/ou idade gestacional ≤32 semanas, sem malformações congênitas. O instrumento eletrônico foi desenvolvido com base em planilhas do Microsoft Excel 2010 para calcular automaticamente ganho de peso corporal, calorias e macronutrientes diariamente recebidos pelos pacientes por meio de dietas parenteral e enteral. Para comparar a dieta prescrita e a administrada, foram utilizados os resultados das médias semanais.

**Resultados:**

Para avaliar o instrumento, foram incluídos 60 recém-nascidos com peso de nascimento de 1.289±305 g e idade gestacional de 30±2 semanas. Destes, 9,6% apresentavam restrição de crescimento no nascimento e 55% no momento da alta. A média de internação foi de 45±17 dias. Foram verificadas diferenças significativas entre a dieta prescrita e a administrada para todos os macronutrientes e calorias totais nas três primeiras semanas. O lipídeo foi o macronutriente com o maior erro percentual na primeira semana.

**Conclusões:**

O emprego de uma rotina computacional foi importante para verificar discrepâncias entre a dieta prescrita e a administrada. Essa análise é necessária para minimizar erros de cálculo e agilizar as decisões da equipe de saúde acerca da abordagem nutricional, podendo contribuir para a segurança do paciente e para a boa prática nutricional. Os recém-nascidos de muito baixo peso são extremamente vulneráveis às deficiências nutricionais e qualquer redução nos macronutrientes recebidos pode ser deletéria para o crescimento satisfatório.

## INTRODUCTION

Adequate neonatal nutrition greatly influences children’s growth and development, impacting the incidence of chronic noncommunicable diseases in adults.^1^ ^,^ ^2^ Postnatal restricted growth is a problem to be studied and solved in most Neonatal Intensive Care Units (NICUs).^3^ ^,^ ^4^ ^,^ ^5^ ^,^ ^6^ ^,^ ^7^ Therefore, monitoring the nutritional adequacy of what was prescribed and what was effectively administered is one of the fundamental elements of care quality.

Studies aiming to make the nutrition given the same as the prescribed nutrition are essential in the handling of preterm infants, in which small differences between the two diets result in undesirable outcomes. In addition, calculations done on newborns with weights that are in the decimals are also subject to error. Thus, it is necessary to develop an instrument that allows for the calculation of the difference between the prescribed (planned) nutrition and what was actually administered, so as to minimize the possibility of calculation error, as well as to improve the quality of the results.

Gnigler et al.^8^ created a spreadsheet in Microsoft Excel 2010 that facilitates the calculation of the parenteral nutrition that will be offered. A comparison between the growth data of extremely low birth weight infants born in the two years prior to the implementation of the spreadsheet and those born in the two years following, showed that the latter presented better nutritional status and other neonatal care indicators as well as less time using parenteral nutrition.

The objective of this study was to develop an electronic instrument to analyze the adequacy of the prescribed diet and the diet administered during newborn nutritional therapy.

## METHOD

The electronic instrument used to analyze prescribed and administered nutritional therapy for in-hospital infants was developed based on Microsoft Excel 2010 software spreadsheets. The objectives were to standardize and optimize the recording of collected nutritional information, taking into account the current weight of the newborn: volume and concentration of venous hydration with glycated serum, as well as the type and volume of the enteral diet. Different tabs were created for entering data from the daily prescribed and administered diet. All of the parameters could be inserted into the prescribed tab and the administered tab (which was actually infused). With this information, the electronic instrument calculated the prescribed daily and weekly values of total calories and macronutrients (protein, lipids and carbohydrates) for each patient.

The instrument was created in order to calculate the daily intake by current weight, and was composed of six items:

- Total parenteral nutrition (TPN) - both prescribed and administered.
- Venous hydration (VH).
- Oral diet.
- Daily results.
- Weekly results.
- Charts.

The original coloring of the fields in the worksheet indicate their purpose: whites are to be filled in, blues are automatically filled in by the computational routine, yellows contain verification warnings, while greens show results of calculations performed based on entered values.

**Figure 1:**
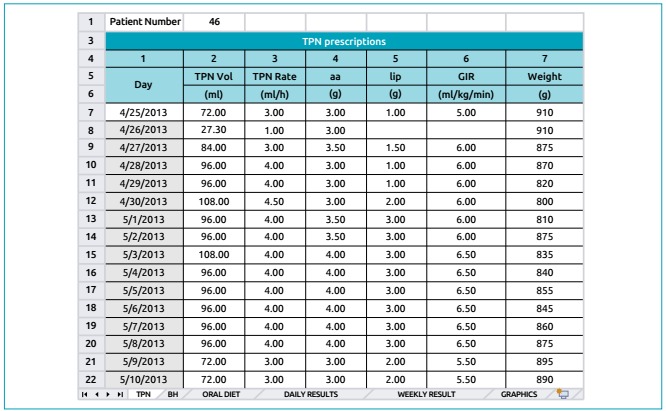
Initial screen of the computational routine. On the left, we observe the prescribed values of total parenteral nutrition (TPN), amino acids (aa), lipids (lip) and glucose infusion rate (GIR) collected in the medical prescription form (prescribed diet). The date of birth and the daily weight of the newborn can also be verified. The first date was entered by the operator, while the instrument completed the cells below. In the lower part of the figure, the follow tabs were in the instrument: TPN, Venous hydration (BH), oral diet, daily result, weekly result and charts.

Figure 1 shows part of the initial screen of the computational routine (TPN tab), which corresponds to the TPN prescriptions. These values came from the medical prescription. Dates and daily weights of the newborns also need to be included.

**Figure 2:**
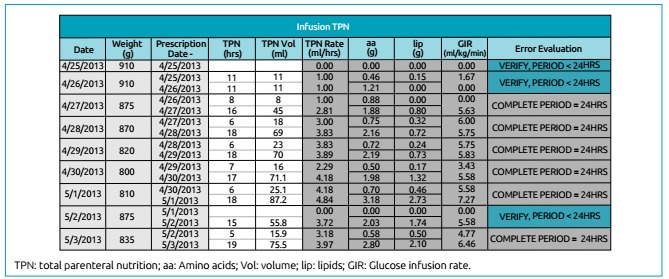
Infusion of total parenteral nutrition. The period (hours) and volume (mL) values are derived from the nursing infusion diary (administered diet). The infusion rate (mL/h), protein (g/kg), lipid (g/kg) and glucose infusion rate (mg/kg/min) presented are calculated by the instrument based on the data of the prescription of total parenteral nutrition (typed on the initial screen).

In Figure 2, TPN infusion data are shown. The values of time (hours) and volume (mL) come from the nursing infusion diary. Infusion rate (mL/h), protein (g/kg), lipid (g/kg), carbohydrate (g/kg) and glucose infusion rate (mg/kg/min) are calculated by the instrument based on the TPN prescription data and the amount infused.

Similar prescription and infusion tables also exist in the VH tab. The glucose infusion rate (mg/kg/min) administered by the prescribed glucose serum concentration and the infusion rate (mL/h) of that solution is calculated. The instrument calculates the data displayed in the green cells. The “Error Evaluation” column shows whether the 24-hour period has been filled in correctly (green) and/or if there is an inconsistency in the results (yellow). If the sum of the hours exceeds 24 hours, a red warning will appear.

In the final tabs, there are the quantitative result of the nutritional approach prescribed and administered per day (daily results tab) and per week of hospitalization (weekly outcome tab), as well as representative charts of one patient. The proteins and calories consumed and the weight gained are expressed as a function of the hospitalization weeks (tab charts).

The nutritional value references for the different types of milk can be changed at any time, so that values of macronutrients dosed in the maternal milk can be included. As a reference for the calculations related to human milk in this study, the values obtained using spectrophotometry (MilkoScan Minor 104, FOSS NIRSystems, Inc., Hillerod, Denmark) were used. The enteral diet consisting of a formula specifically for preterm newborns was calculated based on the information contained on the label of the products used, respecting their volume and dilution.

In order to test the developed instrument, a quantitative, observational and prospective approach was carried out on all of the newborns admitted between May 2014 and December 2016 at the NICU of the Fernandes Figueira National Institute for Women, Children and Adolescents’ Health (IFF), of the Oswaldo Cruz Foundation (Fiocruz), with a birth weight of less than or equal to 1,500 g and/or a gestational age of less than or equal to 32 weeks, and they did not have congenital malformations, genetic syndromes confirmed by a geneticist, or clinically and laboratory confirmed congenital infections. The newborns who presented necrotizing enterocolitis and grades III and IV intracranial hemorrhages were excluded from the study. Data from the first three weeks of hospitalization were analyzed.

Restricted growth was assessed at birth and at hospital discharge, and was considered to be present if the newborn had a Z score for gestational age of ≤-2.0 standard deviations, using the Fenton & Kim curve.^9^

All of the newborns included in this study received nutritional therapy according to the nutritional protocol developed at the studied NICU. This protocol aims to infuse parenteral nutrition hours after birth for all newborns weighing <1,500 g with at least one amino acid. Enteral nutrition is initiated in the first 24‒72 hours of life for all neonates (except for those with intestinal diseases or those that are very unstable). The first choice for milk was colostrum or pasteurized breast milk.

Data from the prescribed diet were obtained from the standardized form showing nutritional evolution, which was completed by the medical team and attached to the medical record. The volume and type of diet administered were calculated for each day the newborn was hospitalized, based on the standardized document and completed by the nursing team. With this information, the nutrition team inserted the macronutrient values (proteins, lipids and carbohydrates) and total calories into the electronic instrument, considering all of the enteral and parenteral routes. To compare the prescribed and administered diet, the results from the weekly averages were used. The results of the analyses were attached to the medical chart to be evaluated by the multidisciplinary team.

Data analysis was performed using the Statistical Package for Social Sciences (SPSS for Windows, Version 20.0, IBM Corp., Armonk, NY, USA). The paired Student’s *t* test was applied to verify the difference between the weekly calorie and macronutrient averages of the prescribed and administered diets. In turn, the Wilcoxon Signed Rank test compared the prescribed and administered values. The difference between prescribed and administered values was also calculated as an error percentage. Differences were considered to be a significant if the value of p <0.05.

This study is a subproject of the study called Perinatal Period Disorders and Their Consequences on Growth, Development and Body Composition of Preterm Newborns: A Cohort Study, approved by the Research Ethics Committee of the IFF (Protocol of Certificate of Presentation for Ethical Evaluation, *Certificado de Apresentação para Apreciação Ética* - CAAE nº 00754612.9.0000.5269). The research was conducted according to Resolution number 466/2012, of the National Health Council (*Conselho Nacional de Saúde* - CNS), and newborns were included after their legal representatives authorized and signed the free and informed consent forms.

## RESULTS

During the study period, 158 newborns weighing less than 1,500 g and who had a gestational age of less than 32 weeks, were born. Of these, 49 had a congenital malformation, 12 developed necrotizing enterocolitis and 14 had intracranial hemorrhages. In addition, there were 20 deaths and three refused to participate in the investigation. Thus, to test the developed instrument, 60 preterm newborns were included in the study.

The mean birth weight of the studied group was 1.289±305g and the gestational age was 30±2 weeks. Among these newborns, 9.6% had restricted growth at birth and 55% at discharge. The mean length of hospital stay was 45±17 days.

All of the newborns received parenteral nutrition starting on the first day of life, and this feeding route lasted 8±4.8 days. The time it took to reach a full enteral diet (above 100 kcal/kg/day) was on average 16.0±4.3 days. In the first week, the predominant diet involved parenteral nutrition and pasteurized human milk (100%); in the second, pasteurized human milk (61.6%); and in the third week, pasteurized human milk and formula for preterm newborns (36.6%).

The median protein and calorie rates prescribed in the first week were 2.99 g/kg/day and 63 kcal/kg/day, increasing to 3.16 g/kg/day and 96 kcal/kg/day in the third week.

There were statistically significant differences between the prescribed and administered diets for all of the macronutrients and total calories in the three weeks studied. The biggest error happened in the first week for all nutrients, including total calories. Comparing the weeks, it was found that there was a significantly different error from the first week to the other two. The second and third week did not differ from each other (Table 1).

**Table 1:** A comparison of the percentage difference of the error between the values of the administered diet and the prescribed diet in the first three weeks of hospitalization in the neonatal intensive care unit of the Fernandes Figueira National Institute of Women, Child and Adolescent Health, from the Oswaldo Cruz Foundation.

Nutrients	Week 1	Week 2	Week 3	p-value*
	Median (%) (minimum; maximum)	Median (%) (minimum; maximum)	Median (%) (minimum; maximum)	
Protein	- 6.2 (-11.3; -2.67)	-2.1 (-6.3; 0.4)	-1.4 (-4.4; -0.5)	<0.05
Lipid	-10.7 (-14.3; -8.3)	-3.5 (-8.7; -0.6)	-1.2 (-5.8; -0.3)	<0.05
Carbohydrate	-5.6 (-8.9; -3.9)	-2.8 (-5.9; -0.8)	-1.6 (-5.6; -0.04)	<0.05
Energy value	-6.9 (-10.1; -4.7)	-3.0 (-6.3; -0.8)	-1.2 (-5.8; 0.6)	<0.05

*Values refer to the comparison between the first week and weeks 2 and 3.

It was observed that the error in lipid administration was the highest among the three macronutrients in the first week. The others did not differ from each other. In the second week, the lipid error was greater than protein and carbohydrate errors, and similar to calorie errors. There were no differences between the errors in the third week (Table 1).

The difference between the prescribed and infused diet is shown in Table 2, where it is also possible to see the deficit of each nutrient and calories accumulated in the first three weeks of life.

**Table 2:** The quantity of macronutrients (g/kg/day) and the energy value (kcal/kg/day) prescribed and received, and the difference accumulated each week.

	Weeks		
	1	2	3
Protein			
Prescribed	3.0 (0.1; 3.7)	2.9 (1.2; 4.4)	3.2 (1.1; 4.3)
Received	2.6 (0.1; 3.4)	2.8 (1.2; 3.9)	3.1 (1.4; 4.2)
Difference	-0.3 (-0.9; 0.2)	-0.1 (-0.6; 0.5)	-0.0 (-0.9; 0.9)
Accumulated difference	-0.3 (-0.9; 0.2)	-4.2 (-5.4; -0.6)	-8.2 (-9.8; -5.6)
Lipid			
Prescribed	1.4 (0.1; 2.8)	2.8 (1.5; 4.2)	4.0 (1.3; 6.8)
Received	1.2 (0.1; 2.6)	2.7 (1.5; 5.1)	3.9 (1.1; 6.6)
Difference	-0.2 (-0.5; 0.7)	-0.1 (-0.7; 1.7)	-0.1 (-1.3; 1.2)
Accumulated difference	-0.2 (-0.5; 0.7)	-0.3 (-0.9; 1.9)	-0.3 (-1.6; 3.1)
Carbohydrate			
Prescribed	9.1 (5.6; 13.6)	10.4 (5.7; 14.7)	11.0 (5.7; 16.0)
Received	8.2 (4.7; 12.9)	10.1 (5.6; 14.9)	10.7 (5.3; 15.7)
Difference	-0.9 (-2.7; 1.4)	-0.3 (-1.6; 1.4)	-0.2 (-1.7; 0.6)
Accumulated difference	-0.9 (-2.7; 1.4)	-1.3 (-3.5; 1.1)	-1.5 (-4.4; 0.8)
Energy value			
Prescribed	63.2 (32.8; 94.3)	81.0 (41.3; 102.0)	96.7 (45.5; 131.8)
Received	54.5 (23.5; 89.1)	79.0 (40.5; 100.5)	92.7 (49.2; 131.2)
Difference	-6.8 (-17.1; 2.7)	-2.5 (-15.7; 11.3)	-1.2 (-17.8; 11.0)
Accumulated difference	-6.8 (-17.1; 2.7)	-9.0 (-26.6; 11.4)	-10.4 (-32.5; 22.4)

## DISCUSSION

This is the first Brazilian study using a computational tool to compare diets prescribed and administered in the neonatal period. It was initially designed for research purposes, but in view of its applicability, can also be incorporated into clinical practice. Previously, the registration of diets was done manually, making it error-prone and very time consuming, and thus compromising the verification of data in a timely manner for the health team to make decisions. This time-saving opportunity is reflected in the possibility to verify previously unrecorded calculations, which may have an impact on the quality of care of preterm newborns. The use of electronic data represents contemporary medical management. ^10^

Several authors have demonstrated the benefits of using electronic programs to optimize the prescription of parenteral nutrition safely and effectively in NICUs.^8^ ^,^ ^11^ ^,^ ^12^ ^,^ ^13^ Puancgo et al.^12^ found an improvement in the quality of patient care after the automation of prescribed parenteral nutrition, due to the reduction of repetitive tasks and tedious calculations, which were previously required of neonatologists, nutritionists and pharmacists. Thus, these reports support the present study.

The differences found between the prescribed and the administered diets may be related to the rejection of the diet by the newborn and/or the delay in changing the prescription for the day, using the prescribed diet from the previous day for longer periods. The largest error seen, especially in the first week, was that related to the administration of lipids, which was 10% lower than prescribed. We speculate that this may be due to the fact that solutions containing protein and glucose are more readily available in the neonatal unit, unlike lipids, which need to be added in the solutions at sites that are suitable for parenteral nutrition preparation.

The result of the present study demonstrates the importance of using electronic instruments to evaluate neonatal diets. Gnigler et al.^8^ carried out a study comparing the before and after of using an electronic instrument for the calculation of parenteral nutrition offered to the newborns during hospitalization, and verified an improvement in the nutritional status and neonatal care indicators. Investigations to evaluate the effectiveness of electronic instruments are necessary.

A study developed in critically ill adults showed a 40% difference between the prescribed and administered diet, mainly due to food breaks when procedures were performed, which may compromise the health of the individual. According to the authors, it is essential to reflect on the attempts to minimize the discrepancies between nutritional planning and its effectiveness.^14^

It should be noted that 9.6% of newborns admitted had restricted growth at birth, while at hospital discharge this value increased 5.7-fold, that is, it rose to 55%. This restriction found in our study may be associated with the deficiency in the intake of protein and other nutrients during the first three weeks of hospitalization.^15^ ^.^ ^16^

The nutritional approach in early life is a conditioning factor of current nutritional status and health in the future. According to Poindexter et al., 1698, 1709-201717, 1728, early administration of 3 g / kg / day of protein in the first five days of life provides more adequate growth. It was also observed that the administration of lipids and carbohydrates was also below the prescribed level. Consequently, the calories administered were lower than those planned for the prescription. This discrepancy between prescribed and administered diets may have contributed to the restriction of growth at the time of discharge. In addition, lipid restriction in the first weeks of life may compromise neurodevelopment.^21^ Stoltz Sjöström et al.^22^ showed that low calorie consumption during the first four weeks of life is an independent risk factor for retinopathy of severe prematurity. This implies that an adequate supply of calories through enteral and parenteral nutrition during the first four weeks of life may be an effective method to reduce the risk of restricted growth, improve myelination and cognitive/motor development, and decrease the incidence of retinopathy in prematurity.

The nutritional management of preterm infants presents a challenge. These patients have restricted water capacity and are submitted to diseases capable of altering how they make use of nutrients, which in turn impacts the adequacy of nutritional prescriptions.^23^ ^,^ ^24^ Despite the existence of clinical protocols, there is a variation rate among the intra-prescriptions and between the prescribed and administered values, which can lead to errors and inadequate behaviors, such as suspension of human milk.^25^ In addition, calculations of water and caloric rates may indicate small amounts to be administered, often milliliter fractions, especially in newborns weighing less than 1,000 g. Errors that tend to underestimate or overestimate desirable values potentially have a major impact on the health and growth of preterm newborns.

It can be concluded that the use of a computational routine was important to ascertain discrepancies between prescribed diets and administered diets. This analysis is necessary to minimize calculation errors and to speed up decisions made by the health team regarding the nutritional approach, which can contribute to patient safety and good nutritional practice. Very low birth weight infants are extremely vulnerable to nutritional deficiencies, and any reduction in macronutrients received may be deleterious for satisfactory growth.
